# Distance learning during the COVID-19 pandemic for children with ADHD and/or ASD: a European multi-center study examining the role of executive function deficits and age

**DOI:** 10.1186/s13034-022-00540-4

**Published:** 2022-12-13

**Authors:** Lisa B. Thorell, Anselm B. M. Fuermaier, Hanna Christiansen, Ricarda Steinmayr, Dieter Baeyens, Almudena Giménez de la Peña, Madeleine J. Groom, Iman Idrees, Saskia van der Oord, Barbara J. van den Hoofdakker, Marjolein Luman, Irene C. Mammarella, Charlotte Skoglund

**Affiliations:** 1 Department of Clinical Neuroscience, Karolinska Institutet, Nobels Väg 9, 17177 Stockholm, Sweden; 2 University of Groningen, Groningen, The Netherlands; 3 Philipps University, Marburg, Germany; 4 TU Dortmund, Dortmund, Germany; 5 KU Leuven, Leuven, Belgium; 6 University of Málaga, Malaga, Spain; 7 University of Nottingham, Nottingham, UK; 8 University Medical Center Groningen, University of Groningen & Accare Child Study Center, Groningen, The Netherlands; 9 Vrije Universiteit Amsterdam, Amsterdam Public Health Research Institute, & Levvel, Specialists in Youth and Family Care, Amsterdam, The Netherlands; 10 University of Padua, Padua, Italy; 11 Uppsala University, Uppsala, Sweden

**Keywords:** Distance learning, COVID-19 pandemic, ADHD, ASD, Executive function deficits, Age

## Abstract

**Background:**

One of the COVID-19 pandemic consequences that has affected families the most is school lockdowns. Some studies have shown that distance learning has been especially challenging for families with a child with neurodevelopmental disorders such as ADHD or ASD. However, previous studies have not taken the heterogeneity of these disorders into account. The aim of the present study was therefore to investigate differences between families with a child with ADHD, ASD, or both conditions, and to examine the role of underlying deficits in executive functioning (EF) in both children and parents in relation to negative and positive effects of distance learning.

**Methods:**

Survey data assessing both negative and positive experiences of distance learning were collected from parents with a child aged 5–19 years in seven Western European countries: the UK, Germany, Spain, Sweden, the Netherlands, Italy, and Belgium. Altogether, the study included 1010 families with a child with ADHD and/or ASD and an equally large comparison group of families with a child without mental health problems. We included measures of three different types of negative effects (i.e., effects on the child, effects on the parent, and lack of support from school) and positive effects on the family.

**Results:**

Results confirmed that families with a child with ADHD, ASD or a combination of ADHD and ASD showed higher levels of both negative and positive effects of distance learning than the comparison group. However, few differences were found between the clinical groups. Group differences were more pronounced for older compared to younger children. Regarding the role of both ADHD/ASD diagnosis and EF deficits, primarily children’s EF deficits contributed to high levels of negative effects. Parent EF deficits did not contribute significantly beyond the influence of child EF deficits. Families of children with ADHD/ASD without EF deficits experienced the highest levels of positive effects.

**Conclusions:**

School closings during COVID-19 have a major impact on children with EF problems, including children with neurodevelopmental disorders. The present study emphasizes that schools should not focus primarily on whether a student has a neurodevelopmental disorder, but rather provide support based on the student’s individual profile of underlying neuropsychological deficits.

**Supplementary Information:**

The online version contains supplementary material available at 10.1186/s13034-022-00540-4.

## Introduction

Previous studies have shown that distance learning during the COVID-19 pandemic had great negative effects on the lives of both children and their parents [e.g., 1, 2]. It has also been suggested that families with children diagnosed with neurodevelopmental disorders such as Attention Deficit Hyperactivity Disorder (ADHD) and Autism Spectrum Disorders (ASD) may have been particularly at risk of adverse effects during the pandemic [e.g., 3–5]. Moreover, there is empirical support for this notion [e.g., 6–10], with managing distance learning being identified as one of the most serious problems for families with a child with ADHD or ASD [e.g., 11–14]. However, previous studies have most often included samples that were too small to investigate group differences between children with ADHD, ASD or a combination of these two disorders. Even more importantly, ADHD and ASD are highly heterogeneous disorders. To better account for the individual needs of children with neurodevelopmental disorders during possible future school lockdowns, it is therefore important to not only focus on diagnostic status, but also on underlying neuropsychological deficits. Both ASD and ADHD have been shown to be strongly related to executive function (EF) deficits [e.g., 15, 16], and EF deficits have been shown to be an important predictor of academic achievement among both students in general [17] and students with ADHD [e.g., 18]. Previous studies have also shown that families with younger children have experienced more problems during the COVID-19 pandemic than have those with older children [e.g., 1, 19]. The overall aim of the present study was therefore to investigate to what extent ADHD/ASD status and EF deficits have additive and/or interaction effects in relation to both negative and positive effects of distance learning during the COVID-19 pandemic and to explore the role of age in these associations.

### Executive functioning, ADHD, ASD, and academic achievement

A considerable body of research has found that children with ADHD [e.g., 16] and ASD [15] perform more poorly compared to controls regarding EF abilities such as inhibition, working memory, and cognitive flexibility. However, when conducting person-oriented analyses rather than simply investigating group differences, it has been shown that only a subgroup of individuals with ADHD [e.g., 20–22] or ASD [e.g., 23, 24] have pronounced EF deficits. Previous research has also shown an association between EF deficits, especially poor working memory, and academic underachievement [e.g., 17, 25].

Distance learning most likely placed higher demands on children’s EF abilities than traditional classroom education does, due to greater working memory demands associated with planning one’s own schoolwork. It also increased demands on inhibition processes to prevent distractions within the home setting, such as computer games and social media. Learning per se may also be more difficult during distance education for students with EF deficits due to the introduction of new teaching platforms and new educational tools. In addition, many children with ADHD and ASD have special educational needs, and research has shown that, during the COVID-19 pandemic, extra educational support from schools was either non-existent or insufficient [e.g., 1, 8, 26–28]. Thus, lack of support from the school may have increased demands on children’s EF during distance learning.

Very few previous studies have investigated the role of EF deficits in distance learning. However, one study of typically developing children [29] showed that children’s EF deficits was a strong positive predictor of negative effects of distance learning on both children and their parents, as well as a modest, although significant, negative predictor of positive effects of distance learning. In addition, Breaux et al. [30] found that EF deficits were associated with a decrease in grade point average (GPA) during the COVID-19 pandemic. Finally, Hai et al. [27] found that EF deficits were related to challenges adjusting to distance learning among children with ADHD, although EF deficits were only briefly assessed using a few single item measures. In summary, a few studies have shown that EF deficits are of relevance to understanding differences in how students have been able to cope with distance learning during the COVID-19 pandemic. However, as previous studies have not examined ADHD/ASD diagnosis and EF deficits as separate predictors, we do not know to what extent effects of distance learning are related to (1) ADHD/ASD diagnosis, (2) EF deficits (i.e., children with EF deficits have experienced negative effects regardless of whether they have ADHD/ASD or not), or (3) an interaction of ADHD/ASD diagnosis and EF deficits (i.e., it is primarily children with both ADHD/ASD and EF deficits who have experienced negative effects).

### Parental influences on the effects of distance learning

Several previous studies have reported that parents found the quality of children’s education during distance learning to be poor [1, 27, 31], with parents of children with ADHD reporting more problems compared to controls [32]. Lack of online teaching, especially for younger children, has resulted in parents being largely responsible for their child’s learning during school closures [e.g. 1]. Consequently, school lockdown combined with distance learning of poor quality has left parents with the primary responsibility of supervising their child’s schooling, often while also trying to carry out their ordinary day-time work. For parents with a child with a neurodevelopmental disorder, this has most likely been especially challenging. One reason for this could be that many children with ADHD and ASD also have academic difficulties and therefore require more extensive parental support compared to children without these diagnoses. This support has included not only providing the child with adequate instructions, but also providing structure and helping the child pay attention and stay motivated. Executive functioning generally improves greatly during middle childhood [e.g., 33], making children increasingly able to take responsibility for their schoolwork by adolescence. However, for children with ADHD and/or ASD, this development is often delayed [34, 35]. Given these findings, one may suggest that only very few children can manage distance schooling well on their own in the lower grades. However, in adolescence, most children in the comparison group, but still only a few among those with ADHD/ASD, can manage well on their own. We would then hypothesize that differences between families with and without ADHD/ASD to be greater for older than for younger children. However, to our knowledge, no previous COVID-19 study has addressed this issue.

Another reason why families with a child with ADHD or ASD might experience more problems during distance learning is that both ADHD [e.g., [36] and ASD [e.g., [37] are highly heritable disorders. Parents therefore often have similar difficulties as their child, which can further amplify problems when parents must take on teaching responsibilities. Previous studies investigating the role of parents during distance learning have found that the association between parental involvement and the child’s difficulties with carrying out distance learning during the pandemic was significantly stronger among families with a child with ADHD compared to controls [38]. In addition, parents’ difficulties with managing distance learning during the pandemic have been shown to be related to increased parental stress [39], and parental quality of life has decreased during the pandemic, especially among parents of children with ADHD and/or ASD [40]. Interestingly, previous research conducted before the pandemic has also shown that parent’s own EF skills are essential to their ability to support their child’s learning [e.g., [41]. Thus, it should be important to also investigate the effect of parent’s EF deficits on distance learning.

### Potential positive effects of school lockdowns

Interestingly, there is evidence that children have experienced positive effects of distance learning, with some studies showing significantly larger positive effects of distance learning for children with mental health problems compared to controls. This has included positive effects such as less bullying and fewer social conflicts [42], more time to complete academic work [14], more positive mood [43], and positive effects in general [1]. Other potential advantages of distance learning, compared to the traditional classroom setting, may be increased flexibility and individualization of learning, provided that the child receives adequate support either from the school or from a parent. Thus, the extent to which distance learning also has had positive effects may vary between children with the same disorder, possibly due to differences in underlying neuropsychological deficits.

### Aims of the present study

The first aim of the present study was to investigate whether there are differences between families with a child with ADHD, ASD, or a combination of ADHD and ASD, and a comparison group without mental health or neurodevelopmental conditions regarding the effects of distance learning during the COVID-19 pandemic. Second, we aimed to investigate whether differences between families with ADHD/ASD and a comparison group vary depending on the age of the child. Third, we aimed to investigate the extent to which ADHD/ASD status and child EF deficits have additive and/or interaction effects in relation to effects of distance learning. Finally, in supplementary analyses, we explored whether parental EF deficits contribute to effects of distance learning beyond the influence of child EF deficits. An increased understanding of the role of underlying neuropsychological deficits may help us understand the mechanisms underlying both adverse and positive outcomes of school lockdowns during the COVID-19 pandemic. More importantly, it may help us tailor interventions and provide targeted support to avoid further negative psychosocial consequences of distance learning and inform preventive strategies to alleviate adverse outcomes during possible future pandemics.

## Methods

### Participants and procedure

The inclusion criterion for the present study was being a parent of a child (5–19 years of age) enrolled in a mainstream school and receiving distance learning due to school closure during the COVID-19 pandemic. If a parent had more than one child receiving distance education, they were asked to rate their oldest child. Altogether, we collected data from 6720 families in Western Europe. From this larger sample, we identified 605 families with a child with ADHD, 207 families with a child with ASD, and 198 families with a child with both ADHD and ASD based on parent reports. Altogether the present study included 1010 families with a child with ADHD and/or ASD from seven European countries: the United Kingdom (n = 125), Sweden (n = 369), Spain (n = 136), Belgium (n = 101), the Netherlands (n = 115), Germany (n = 122), and Italy (n = 42). In addition, we selected a comparison group of families with a child without any known mental health problems or neurodevelopmental condition (n = 1010) who were randomly selected from families who had been matched to the families with a child with neurodevelopmental disorders based on child sex, child age and country. Families were divided into four groups depending on the age of the target child: 1) 5–8 years (n = 230), 2) 9–12 years (n = 580), 3) 13–16 years (n = 600) and 4) 17–19 years (n = 604). Background data for the four groups are presented in Table 1. For further information on the larger sample of 6720 families, please see Thorell et al. [1].

**Table 1:** Results of ANOVAs and Chi-square tests examining group differences with regards to background variable, EF deficits, and effects of distance learning

	Comparison Group (A) n = 1010	ADHD Group(B) *n* = 605	ASD group (C) *n* = 207	ADHD + ASD group (D) *n* = 198	*F*^1^*/χ*^*2*^	Post hoc^1^
Background variables						
Child age, M (SD)	13.44 (3.62)	13.04 (3.62)	13.33 (3.55)	15.11 (3.12)	20.92***	A, B, C < D
Child sex, % boys	63.6	63.3	61.4	67.7	5.65, *ns*	
Ethnicity, (%) foreign background	3.5	3.5	3.0	1.6	2.08, *ns*	
Parent age, M (SD)	45.25 (5.90)	44.77 (6.42)	45.23 (5.85)	45.71 (5.57)	1.45, *ns*	
Parent sex, n (%) mothers	86.2	90.6	90.2	93.9	18.72*	A < B, C, D
Parent education (%)					33.71***	A, C > B
Mandatory schooling only	2.2	4.8	1.0	0.5		
Completed secondary school	11.9	18.1	13.7	19.3		
University education	85.9	77.1	85.4	80.2		
Distance learning (weeks)	7.98 (4.07)	8.36 (3.67)	8.37 (2.90)	8.04 (2.48)	1.58, *ns*	
Executive function deficits, M (SD)						
Children’s EF deficits	2.18 (0.89)	3.71 (0.80)	3.31 (0.91)	3.80 (0.78)	512.49***	A < C < B, D
Parents’ EF deficits	1.78 (0.56)	2.01 (0.67)	1.99 (0.62)	2.11 (0.70)	27.41***	A < B, C, D
Effects of homeschooling, M (SD)						
Negative effects on the child	2.22 (0.90)	2.96 (1.07)	2.76 (1.16)	2.98 (1.18)	82.58***	A < B, C, D
Negative effects on the parent	2.27 (1.18)	3.62 (1.11)	3.29 (1.24)	3.47 (1.20)	85.88***	A < B, C, D; B > C
Lack of support from the school	2.43 (1.01)	3.00 (1.05)	2.82 (1.10)	2.90 (1.10)	39.85***	A < B, C, D
Positive effects on the family	2.80 (1.02)	2.78 (1.14)	3.10 (1.18)	2.94 (1.20)	4.78**	A, B < C

* *p* < .05; **p < .01; ***p < .001

^1^Using Welch’s adjusted *F*-ratio and the Games-Howell post hoc procedure to adjust for unequal variances

Data were collected from April 28 to June 21, 2020, using an anonymous digital survey distributed to parents via social media, schools, parent networks, and parent support groups. Because the overall project focused on neurodevelopmental disorders, families with mental health problems were oversampled in all countries except Germany and Italy. This oversampling was achieved by posting information about the study on various social media forums targeting mental health problems in general or forums or support groups specifically targeting ADHD and/or ASD. The study was approved by the ethics committees in each of the seven participating countries.

### Material

The online questionnaire focused on several aspects of parents’ experiences of distance learning. In the present study, we included four domains: (1) negative effects on children, (2) negative effects on parents, (3) positive effects on the family and (4) support from school. Items were developed based on a previous qualitative study (unpublished data) that examined what aspects of family functioning parents thought were most strongly affected by school closure during the COVID-19 pandemic. In addition, the present study included measures of children’s and parents’ EF deficits. All items were rated on a scale ranging from 1 (“strongly disagree”) to 5 (“strongly agree”), with higher scores indicating more negative effects or greater EF deficits. The measures are described in more detail below.

#### Negative effects on children

The following four items measured parents’ perceptions of negative effects on the child due to distance learning: (1) “Homeschooling puts too high demands on the child to plan his/her own schoolwork”; (2) “My child cannot fully take part in homeschooling and therefore misses some of the school activities” (3) “It is impossible to get homeschooling to work well for my child”; (4) “Homeschooling has had negative effects on my child’s life”. Cronbach’s alpha was 0.80.

#### Negative effects on parents

The following five items measured parents’ perceptions of negative effects for themselves: (1) “As a parent, I need to take an active part in homeschooling to make sure my child is doing the work s/he is supposed to do”; (2) “I feel stressed because of the extra work that homeschooling demands of me as a parent”; (3) “I am worried that my child will not be able to handle school as well as s/he normally does because of homeschooling”; (4) “Homeschooling has had negative effects on my own life”. Cronbach’s alpha was 0.86.

#### Positive effects on the family

The following three items measured parents’ perceptions of positive experiences: (1) “I see some advantages with the fact that my child is homeschooled”; (2) “Homeschooling has had positive effects on my child’s life”; (3) “Homeschooling has had positive effects on my own life”. Cronbach’s alpha was 0.84.

#### Lack of support from the school

The following four items measured parents’ perceptions of support from the school during distance learning: (1) “The information about homeschooling that I have received as a parent from the school has not been sufficient.”; (2) “The school’s support of students during homeschooling is not sufficient. “; (3) “The information provided from the school is not clear enough for my child to carry out his/her studies from home.”; (4) “The quality of my child’s homeschooling is very poor.” Cronbach’s alpha was 0.84.

#### Executive functioning deficits

Children’s EF deficits were measured using an abbreviated (8 items) version of the Childhood Executive Functioning Inventory [CHEXI; 44], and parents’ EF deficits were measured using an abbreviated version (8 items) of the Adult Executive Functioning Inventory [ADEXI; 45]. Both the CHEXI and the ADEXI are freely available in many languages (www.chexi.se), and these rating scales both include two subscales measuring working memory and inhibition. In the present study, we used the mean value for all eight items of the questionnaire. However, sensitivity analyses were also conducted to determine whether the results remained similar when effects of working memory and inhibition were investigated in separate analyses. Cronbach’s alpha, calculated in the present study, was 0.90 for child EF deficits and 0.84 for parent EF deficits.

### Statistical analyses

First, one-way analyses of variance (ANOVAs) for dimensional variables and chi-square tests for categorical variables were used to investigate group differences between ADHD, ASD, ADHD + ASD and the comparison group with regard to background variables, EF deficits and effects of distance learning. Because group sizes were unequal and the assumption of homogeneity of variance was not met (except for parent age and number of weeks of distance learning), we used the Welch’s adjusted *F*-ratio and the Games-Howell post hoc procedure [46]. Effects sizes were calculated using Cohen’s d, where values between 0.20 and < 0.50 were interpreted as a small effect, values between 0.50 and < 0.80 as a medium-size effect and values ≥ 0.80 as a large effect [47]. In case of significant group differences for any of the background variables, ANCOVAs were used to determine whether effects remained similar when including background variables as covariates. Third, and in line with several previous studies [e.g., [22], we defined EF deficits as a score ≥ 90th percentile of the comparison group for both child and parent EF deficits. Two-way ANOVAs, with separate analyses for each of the four outcome measures (i.e., negative effects on children, negative effects on parents, lack of support from school and positive effects), were thereafter used, with one factor representing EF deficits (i.e., EF deficits versus no EF deficits) and the other factor representing parent-reported diagnosis (i.e., ADHD and/or ASD versus comparison group). Effect sizes for the ANOVAs were calculated using partial eta square, where a value of 0.01 indicates a small effect, a value of 0.06 indicates a medium-size effect and a value of 0.14 indicates a large effect [47]. Post hoc analyses were conducted using paired comparisons with Bonferroni correction.

## Results

Table 1 presents the results of the ANOVAs investigating group differences with regard to background variables, EF deficits, and effects of distance learning. With regard to group differences for effects of distance learning, the results of the ANOVAs indicated that families with a child with a neurodevelopmental disorder (i.e., ADHD, ASD, or a combination of both) reported significantly higher negative effects compared to the matched comparison group with regard to negative effects on the child, negative effects on the parent, as well as lack of support from school. No significant group differences were found between the three clinical groups in these domains, except that parents in the ADHD group reported slightly higher levels of negative effects on parents than did parents in the ASD group. Regarding positive effects, a significant main effect of group was found, although with a small effect size. Post hoc analyses revealed that it was the ASD group who reported significantly higher levels of positive effects compared to both the comparison group and the ADHD group. However, effect sizes for these comparisons were small (both *d*s = 0.28). Because all significant group differences between the three clinical groups were very small, these groups were combined in the following analyses. None of the findings reported above changed when conducting ANCOVAs with child age, parent sex, and parent education as covariates.

### Effects of age

**Fig. 1:**
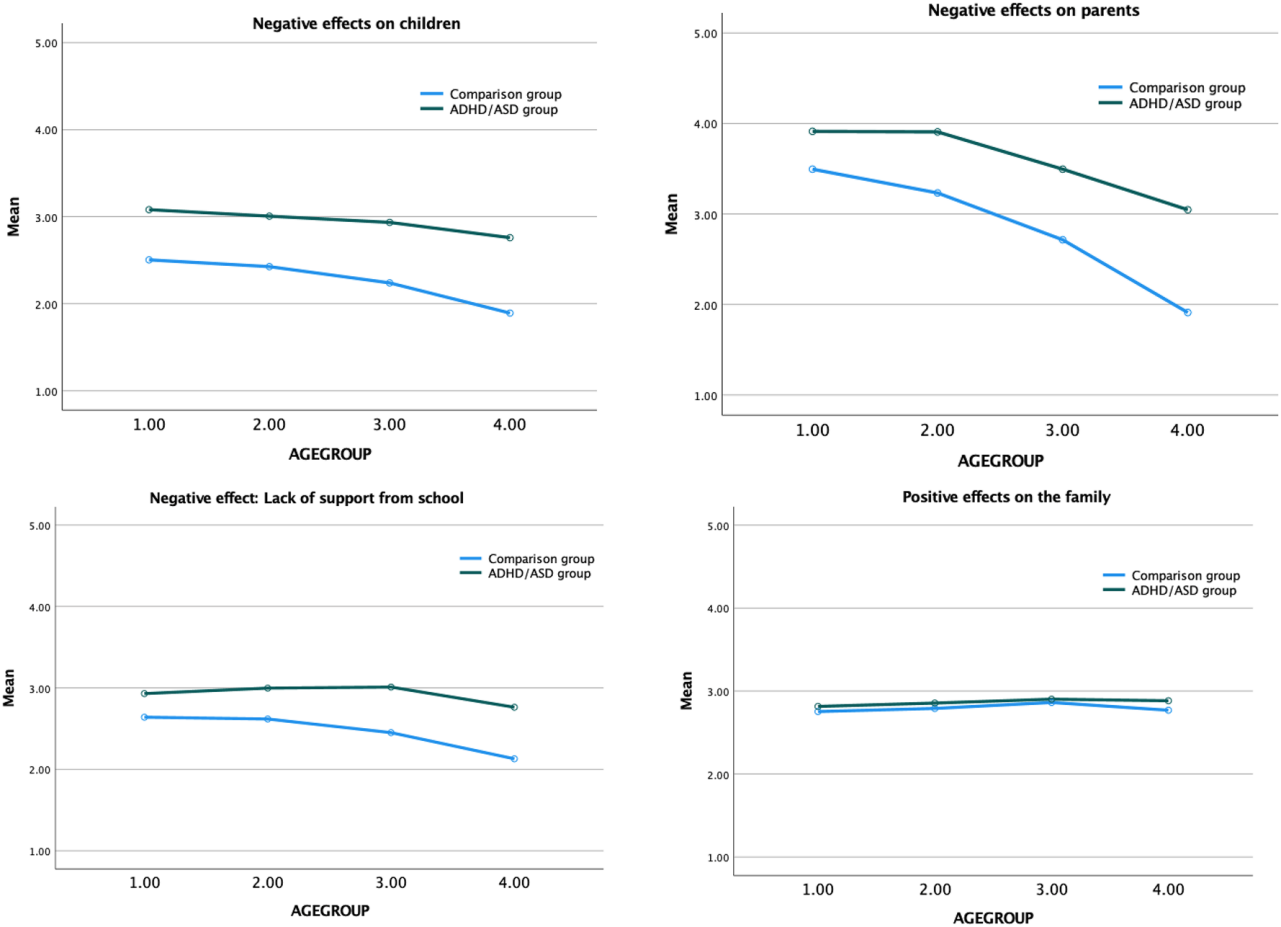
Results of analyses examining the effects of diagnosis and age group

Next, we examined whether group differences between families with and without a child with a neurodevelopmental disorder varied depending on the child’s age. The results showed that there was a significant interaction effect of diagnosis and age for negative effects on parents, *F* = 8.08, *p* < 0.001, as well as marginally significant interaction effects for negative effects on children, *F* = 2.41, *p* = 0.06, and lack of support from school, *F* = 2.49, *p* = 0.06. As shown in Fig. 1, negative effects were larger for families with younger compared to older children in both groups. However, the decrease in negative effects across age was larger for families with ADHD/ASD compared to those without ADHD/ASD. Consequently, with regard to negative effects, group differences between ADHD/ASD versus controls were larger for families with older compared to younger children. The interaction effect of diagnosis and age was not significant for positive effects on the family, *F* = 0.12 *p* = 0.95 (see Fig. 1).

### Effects of EF deficits

In the next step, we conducted a set of 2 × 2 ANOVAs. As described in the methods section, the two factors were neurodevelopmental disorder and EF deficits. Among families with a child with a neurodevelopmental disorder, 68% (66% of those with ADHD, 48% of those with ASD, and 74% of those with ADHD + ASD) had EF deficits.

**Fig. 2:**
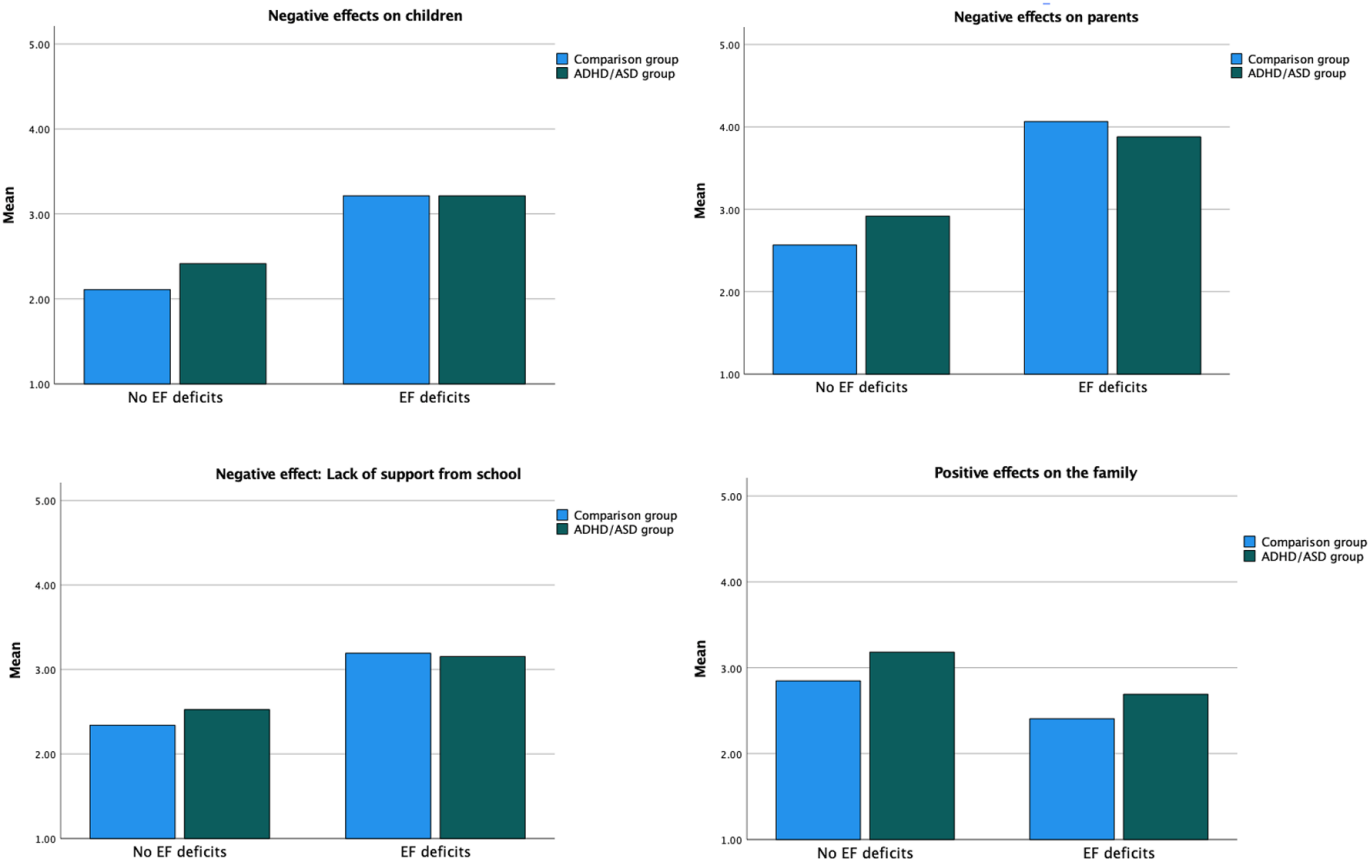
Results of analyses examining the effects of child EF deficits and diagnosis

The results of the two-way ANOVAs (see Table 2 and Fig. 2) showed that the main effect of diagnosis was significant for negative effects on children and positive effects on the family, but not for effects on parents and lack of support from the school. More specifically, families with a child with a neurodevelopmental disorder scored significantly higher with regard to both negative effects on the child and positive effects on the family. However, all effect sizes, even for the significant effects, were small. Regarding the main effects of EF deficits, significant effects were found for all four outcomes measures, with a large effect size for negative effects on the parent, medium effect sizes for the effect on the child and lack of support from the school, and a small effect size for positive effects on the family.

**Table 2:** Results of the ANOVA investigating main effects of diagnosis and child EF deficits, as well as interaction effects of diagnosis and EF deficits

	Neurodevelopmental disorder		Comparison group		Main effect Diagnosis *F* (η^p^)	Main effect EF deficits *F* (η^p^)	Interaction effect Diagnosis x EF deficits *F* (η^p^)
	EF deficits (n = 636) M (SD)	No EF deficits (n = 374) M (SD)	EF deficits (n = 100) M (SD)	No EF deficits (n = 910) M (SD)			
Effects of homeschooling							
Negative effects on the child	3.21 (1.06)	2.42 (1.02)	3.21 (0.93)	2.11 (0.82)	6.57** (< .01)	260.72*** (.12)	6.89** (< .01)
Negative effects on the parent	3.88 (1.00)	2.91 (1.16)	4.06 (0.84)	2.57 (1.12)	1.34 (< .01)	339.12*** (.14)	15.29*** (< .01)
Lack of support from the school	3.15 (1.05)	2.52 (0.96)	3.19 (0.91)	2.34 (0.99)	1.29 (< .01)	141.99*** (.07)	3.16 (< .01)
Positive effects on the family	2.69 (1.14)	3.18 (1.14)	2.41 (1.06)	2.85 (1.01)	21.24*** (.01)	48.09*** (.03)	0.14 (< .01)

**p < .01; ***p < .001

In terms of interaction effects, the results showed that significant interaction effects of diagnosis and EF deficits were found for negative effects on the child and negative effects on the parent, although both with small effect sizes. Post hoc analyses (see Fig. 2) indicated that scores for negative effects on the child and the parent were significantly higher in the ADHD/ASD group than in the comparison group if the child did not have EF deficits. However, no significant group differences were found between the ADHD/ASD group and the comparison group if the child had EF deficits. The results of the sensitivity analyses showed that although the effect of working memory was somewhat stronger than the effect of inhibition with regard to all four outcomes measures, the main findings were very similar to those described above. In addition, none of the findings reported above changed when conducting ANCOVA:s with child age, parent sex, and parent education as covariates.

Finally, we conducted supplementary analyses to determine whether parental EF deficits played a role, beyond the influence of child EF deficits. The results showed that there was a relatively large overlap between parent and child EF deficits. Among families with a neurodevelopmental disorder, 34.5% of those with child EF deficits also had parent EF deficits and 17.7% had parent EF deficits only. In the comparison group, 29.6% of families with a child with EF deficits also had parent EF deficits and 7.7% had parent EF deficits only. When conducting a 2 × 4 ANOVA with diagnosis as the first factor and EF deficits as the second factor comprising 4 levels (i.e., no EF deficits, only parent EF deficits, only child EF deficits and both parent and child EF deficits), the results (see Additional file 1) showed that, for negative effects (i.e., effects on the child, effects on the parents and lack of support), the group with only child EF deficits and those with both child and parent EF deficits had significantly higher scores (indicating more negative effects) compared to those with no deficits and only parent EF deficits. However, those with child EF deficits and both child and parent EF deficits did not differ from one another, and the groups with no EF deficits and only parent EF deficits also did not differ. In terms of positive effects, families with only child EF deficits reported lower levels of positive effects compared to those with only parent EF deficits and those with no EF deficits, but no other significant group differences were found for positive effects. In summary, the supplementary analyses indicated that parent EF deficits had very limited effects on distance learning over and above child EF deficits.

## Discussion

The overall aim of the present study was to investigate both negative and positive effects of distance learning during school closures due to the COVID-19 pandemic for families with a child with ADHD and/or ASD and to explore the role of EF deficits and age in contributing to these effects. A first finding of the present study was that families with a child with ADHD and/or ASD experienced more negative effects on both parents and children, greater lack of support from schools, as well as greater positive effects during distance learning. Differences between families with ADHD, ASD, or the combination of ADHD and ASD were all very small, and mostly non-significant, except for significantly lower negative effects on parents in the ASD group compared to the ADHD group. Differences between the ADHD/ASD group and the comparison group increased with age for negative effects. When investigating the role of child ADHD/ASD status and EF deficits, the results showed that the main effects of diagnosis were small, although significant for negative effects on the child and lack of support from school. However, more importantly, the main effect of child EF deficits was significant for all outcomes with medium or large effects sizes for negative effects on the child, negative effects on the parent and lack of support from school. Finally, supplementary analyses showed that parent EF deficits played a very limited role over and above the effect of child EF deficits.

### Negative effects of distance learning for children with neurodevelopmental disorders

Our finding that families with a child with ADHD and/or ASD experienced more negative effects of distance learning compared to the comparison group was not surprising and was in line with results from several other studies. More specifically, previous research has shown that grade point average (GPA) decreased significantly from academic year 2019–2020 to 2020–2021 for children with ADHD, but not for the comparison group [30]. In addition, it has been shown that families with a child diagnosed with ADHD experienced more difficulties for children and parents had less confidence in supporting their child with distance learning [11]. Thus, there is reason to believe that distance learning during the pandemic had serious negative effects, with greater effects for families with a child with a neurodevelopmental disorder compared to the comparison group. The present study added new information by showing that group differences increased with age. This finding is in line with our hypothesis that older children in the comparison group develop self-regulatory that are needed to take responsibility for their own schooling, whereas this development is delayed among those with ADHD and /or ASD [34, 35].

The present study also added valuable new information by showing that there were few significant group differences between children with ADHD, ASD, and a combination of ADHD and ASD. However, families with a child with ASD—compared to families with a child with ADHD—appear to have managed slightly better, with significantly lower negative effects on parents and significantly higher levels of positive effects. These small, but significant, differences may be a result of the fact that children with ASD without intellectual disabilities have been shown to perform better academically compared to children with ADHD [e.g., 48] and may feel less anxious when at home due to fewer pressures to interact and reduced sensory stimulation [49]. The ADHD group also had parents with significantly lower educational level compared to the ASD group, but the results of the ANCOVAs showed that group differences remained significant when including parent education as a covariate.

Even though significant group differences were found for all four outcome variables when comparing ADHD and/or ASD groups with the comparison group, a different picture emerged when EF deficits were included in the analyses. The results clearly showed that, although the main effect of diagnosis was significant for both negative effects and positive effects on the child, the effect size for EF deficit was substantially higher. This finding is in line with current theoretical models, emphasizing that ADHD and ASD should be regarded as being related to multiple neuropsychological deficits [e.g., 20, 21, 24], as well as the Research Domain Criteria (RDoC) initiative, which emphasizes that we should focus on underlying mechanisms rather than overt symptom levels [e.g., 50]. Regarding practical implications, our findings further emphasize that EF deficits appear to be of central importance for academic under-achievement and there are several available classroom-based interventions that target EF deficits [e.g., 51]. Unfortunately, there is a great need for more empirical research to determine what school-based accommodations that are effective [52] and it has become even more evident during distance learning due to the COVID-19 pandemic that the support provided by the schools need to be better adapted to the needs of individual students, regardless of diagnosis.

### Positive effects of distance learning for children with neurodevelopmental disorders

In line with previous research investigating either ADHD [14] or ASD [43], the results of the two-way ANOVA showed a main effect of diagnosis for positive effects of distance learning. The present study also adds new information by showing that positive effects were primarily linked to having a diagnosis of ASD rather than ADHD. It is possible positive effects were related to the ability to adapt the school day according to individual needs and the ability to reduce stimulus overload during distance learning. This is likely of most importance for students with ASD, as the diagnostic criteria for this disorder include rigid thinking patterns and hypersensitivity to sensory input [53]. Previous research has also shown that school closures have led to increased sleep [14], with one study showing that the proportion of adolescents meeting the sleep recommendations of 8 h/night increased from 13.4% to 37.5% [54]. It is well-known that sleep problems are linked to neurodevelopmental disorders [55, 56], with especially consistent links being found to ASD [57]. Sleep has also been shown to be associated with executive functioning, especially among individuals with high levels of attention problems [e.g., 58]. Thus, increased levels of sleep might have contributed to better academic performance during school closures, with greater effects for children with ADHD/ASD compared to controls.

The present study also contributes new findings by showing that, in addition to a main effect of having an ADHD/ASD diagnosis, it was primarily families with a child with ADHD/ASD without EF deficits that reported higher levels of positive effects. Thus, it is possible that adequate EF skills are required to be able to take advantage of the increased flexibility associated with distance learning. When a child has EF deficits, the increased flexibility may instead provide too little structure, which increases the risk that the child will get distracted by other things in the home setting, such as computer games and social media [e.g., 59, 60]. Previous studies conducted during the COVID-19 pandemic have also shown significant associations between digital media use and mental health outcomes [61, 62].

In addition to the possible positive effects mentioned above, it should be considered important to generate further knowledge concerning whether the positive effects reported for children with mental health problems are truly positive effects of distance learning or whether such effects are better explained as an absence of the negative effects of regular schooling. A considerable body of research conducted before the pandemic has shown that children with ADHD and/or ASD do not only perform worse academically, but that they, more often than controls, experience school-related problems such as higher levels of bullying [e.g., 63, 64], school anxiety [49], lower school well-being [65], more peer problems [e.g., 66, 67], as well as lower social involvement in the classroom [e.g., 68]. In addition, studies conducted during the COVID-19 pandemic have revealed a decrease in, for example, bullying [e.g., 69] and body-weight concerns [70]. Thus, it is likely that the positive effects we see in the present study are at least partly related to the fact that children with ADHD/ASD are less satisfied with school, and distance learning meant that they did not have to deal with the social pressure associated with attending school.

### Strengths and limitations

A major strength of the present study was its large sample size, including over 2000 families recruited from seven different European countries. This gave us enough power to also compare different clinical subgroups with one another, something that has not been done in previous studies. It should also be considered a strength that ratings were collected during school lockdowns rather than relying on retrospective reports, as has been done in many previous studies. Another strength was that we considered both ADHD and ASD as heterogeneous disorders and examined subgroups based on underlying EF deficits. Moreover, we assessed EF deficits in both the child and the parent. Regarding limitations, we relied on parents’ judgements of their current functioning compared to pre-pandemic functioning rather than collecting data before the pandemic. Because we used anonymous surveys, we could not confirm the diagnoses of the children using medical records and this also made it impossible to collect follow-up data during a later phase of the pandemic. The lack of follow-up data may have underestimated the effects, as negative effects most likely increased during the course of the pandemic. It should also be regarded as a limitation that our survey did not contain information about the COVID-related restrictions in the different countries/regions, or more detailed questions on how distance learning was carried out. Finally, the present study only assessed effects of EF deficits using parent ratings, without including neurocognitive testing, and we did not include other neuropsychological deficits that have been shown to be related to ADHD and/or ASD such as emotional and motivational functioning (i.e., delay aversion). A previous study showed that children with ADHD and poor emotion regulation showed the largest increase in externalizing, but not internalizing, symptoms during the pandemic [7]. However, we chose to focus on EF deficits, as they have been shown to be of most relevance for academic outcomes [71].

## Conclusions

The results of the present study show that for all four outcome measures, which included both positive and negative effects of distance learning, having EF deficits played a greater role than having an ADHD/ASD diagnosis. This emphasizes that schools should not focus primarily on whether a student has a neurodevelopmental disorder, but rather provide support based on the student’s individual profile of underlying neuropsychological deficits.

## Supplementary Information

**Additional file 1.** Results of the ANCOVA:s investigating the effect of both child and parent executive function deficits on distance learning.

## Data Availability

The datasets used and/or analyzed during the current study are available from the corresponding author on reasonable request.

## Abbreviations

ADHD: Attention deficit hyperactivity disorder
ANOVA: Analysis of variances
ASD: Autism spectrum disorders
EF: Executive functioning
