# Supplementary material for: Distance learning during the COVID-19 pandemic for children with ADHD and/or ASD: a European multi-center study examining the role of executive function deficits and age
Source: Child Adolesc Psychiatry Ment Health. 2022 Dec 13;16:101. doi: 10.1186/s13034-022-00540-4 (PMC9745722; doi:10.1186/s13034-022-00540-4)

**ADDITIONAL FILE 1**

**Distance learning during the COVID-19 pandemic for children with ADHD and/or ASD: A European multi-center study examining the role of executive function deficits and age**

In the supplementary analyses, we conducted a 2 x 4 ANOVA with diagnosis as the first factor (i.e., ADHD/ASD versus comparison group) and EF deficits as the second factor (i.e., no deficits, only parent EF deficits, only child EF deficits, and both child and parent EF deficits). The results showed that there was a significant effect for negative effects on children, *F* = 4.45, *p* < .05 and positive effects, *F* = 12.90, *p* < .001. However, all effects of diagnosis had a small effect size. In addition, there was significant effects of EF deficits, with regard to negative effects on children, *F* = 88.19, *p* < .001, negative effects on parents, *F* = 114.58, *p* < .001, lack of support from school, *F* = 47.40, *p* < .001 and positive effects, *F* = 16.71, *p* < .001. Effect sizes were large for negative effects in parents, medium for negative effects on children and lack of support from school, and small for positive effects.

Post hoc analyses (see Figure 3) showed that for the three types of negative effects, the group with only child EF deficits and those with both child and parent EF deficits had significantly higher scores (indicating more negative effects) compared to those with no deficits and only parent EF deficits. Families with child EF deficits and both child and parent EF deficits did not differ from one another, and the groups with no EF deficits and only parent EF deficits also did not differ. For positive effects, families with only child EF deficits reported lower levels of positive effects compared to those with only parent EF deficits and those with no EF deficits, but no other significant group differences were found. In summary, the supplementary analyses indicated that parent EF deficits had very limited effects on distance learning over and above child EF deficits.

**Figure 3**

*Graphs displaying the effects of diagnosis and parent/child EF deficits for the four outcome measures.*


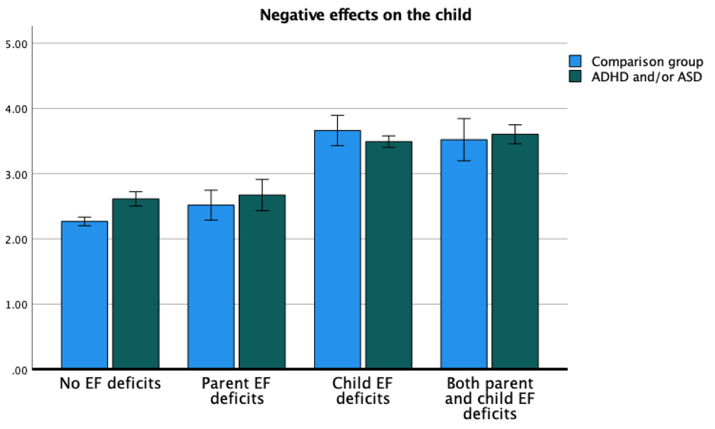

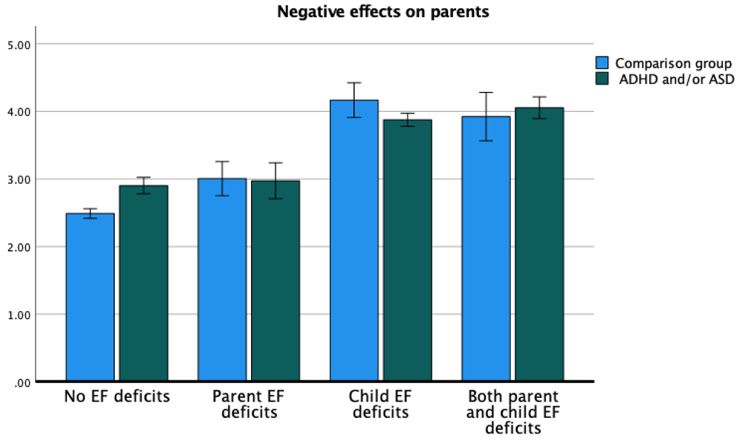


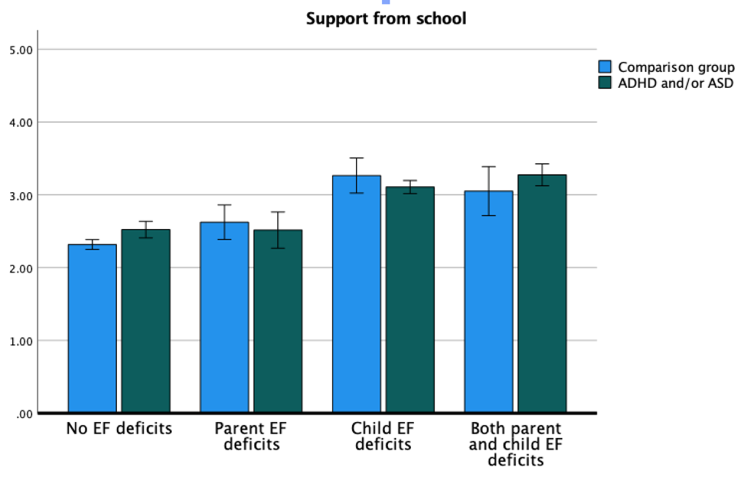

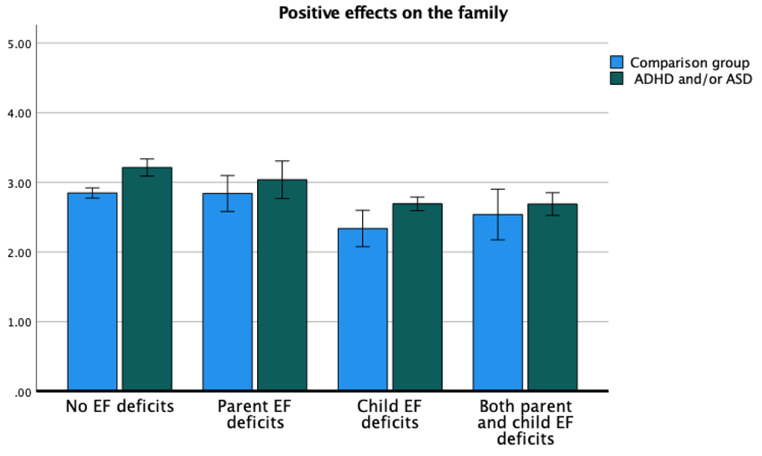

Supplement: Supplementary file 1 — Additional file 1. Results of the ANCOVA:s investigating the effect of both child and parent executive function deficits on distance learning. [file 13034_2022_540_MOESM1_ESM.docx]
